# Correction: Martinez et al. Cavitation Characterization of Size-Isolated Microbubbles in a Vessel Phantom Using Focused Ultrasound. *Pharmaceutics* 2022, *14*, 1925

**DOI:** 10.3390/pharmaceutics14102246

**Published:** 2022-10-21

**Authors:** Payton Martinez, Nick Bottenus, Mark Borden

**Affiliations:** 1 Biomedical Engineering Program, University of Colorado, Boulder, CO 80309, USA; 2 IQ Biology Program, University of Colorado, Boulder, CO 80309, USA; 3 Mechanical Engineering Department, University of Colorado, Boulder, CO 80309, USA

## Error in Figure

In the original publication [1], there was a mistake in Figure 4 as published. Figure 3 was published twice in lieu of Figure 4. The corrected Figure 4 appears below. The authors state that the scientific conclusions are unaffected. This correction was approved by the Academic Editor. The original publication has also been updated.

## Figures and Tables

**Figure 4:**
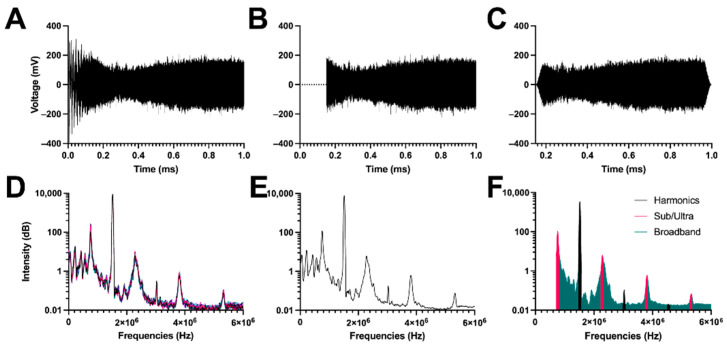
Signal processing of voltage versus time data obtained from the passive cavitation detector for 1.5 × 10^6^ MBs/mL of 5 µm of MBs sonicated at 1.0 mechanical index. (**A**–**F**) Similar signal processing as Figure 3.
