# Supplementary material for: Cavitation Characterization of Size-Isolated Microbubbles in a Vessel Phantom Using Focused Ultrasound
Source: Pharmaceutics. 2022 Sep 12;14(9):1925. doi: 10.3390/pharmaceutics14091925 (PMC9501432; doi:10.3390/pharmaceutics14091925)
Supplement: Supplementary file 1 [file pharmaceutics-14-01925-s001.zip › pharmaceutics-1887195-supplementary.pdf]

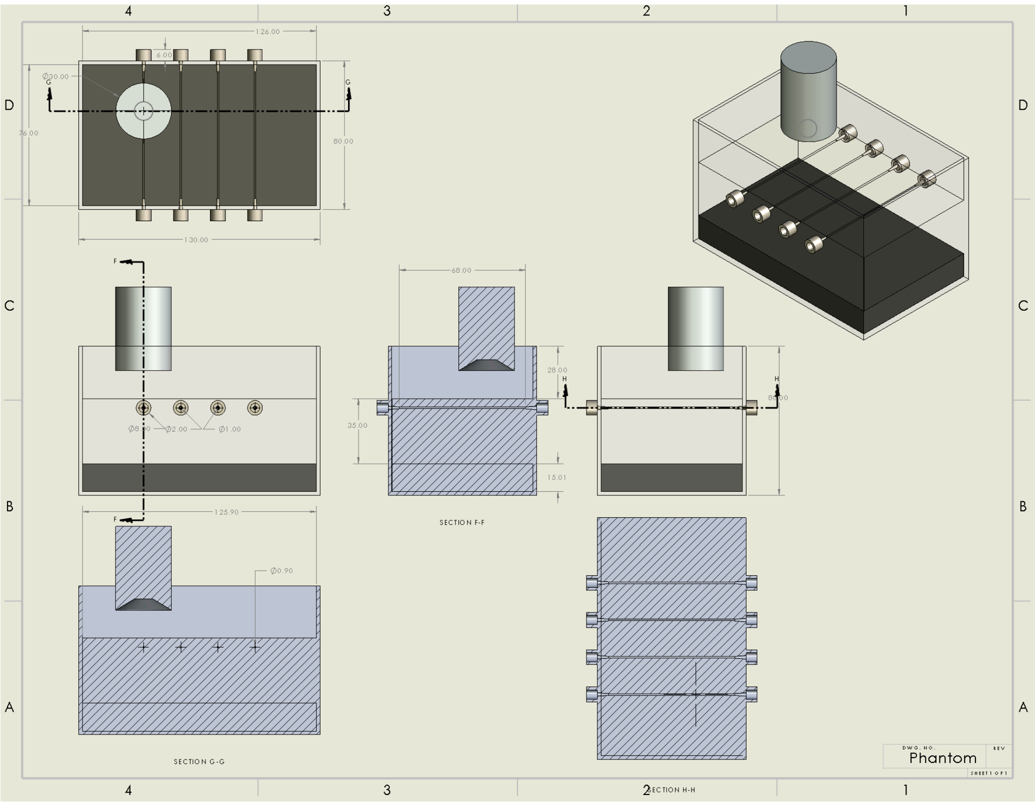

Figure S1. Phantom Model Box. This is an illustration of the modeled phantom box used during the study. Dimensions are labeled in SI units. Grey diagrams are slice views that are sliced at black dotted lines shown in non grey diagrams. Diagrams also show FUS transducer to orient viewer.

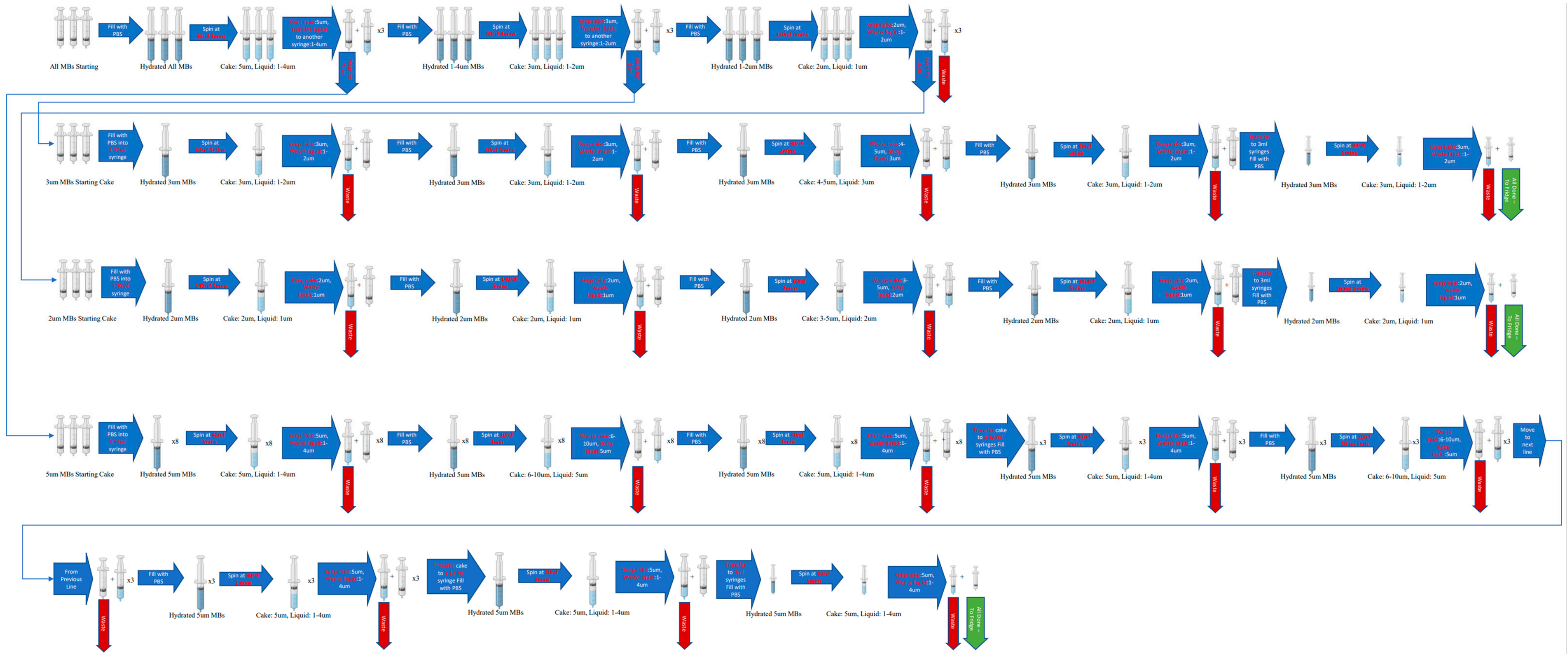

Figure S2. Size Isolation of Monodisperse Microbubbles. This is an illustration of the process used to isolate the three size distributions used in the study. Thicker blue arrow illustrates separation of phases or centrifugation. Green arrows show when cake was to be saved for measuring.

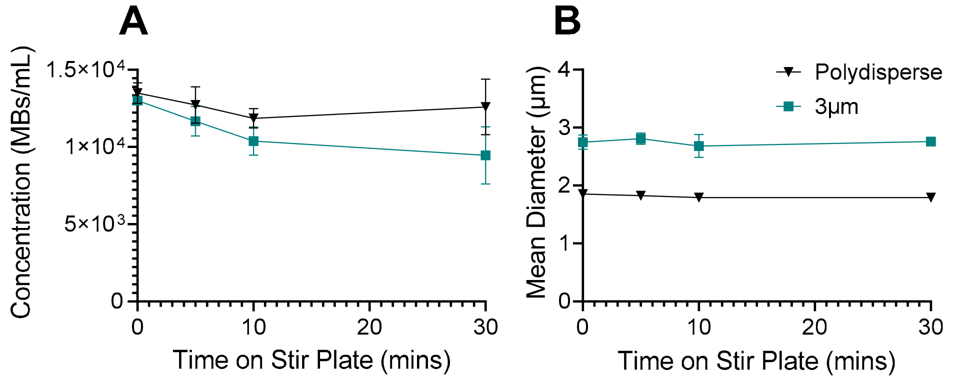

Figure S3. Microbubble Stability During Stirring. (**A**) Graph illustrates the change in concentration as the microbubbles are stirred. Graph (**B**) shows the change in mean diameter of the number weighted size distributions. Concentrations are the lowest concentrations used in this study. Data represent the mean and error bars show standard deviation (N = 3).

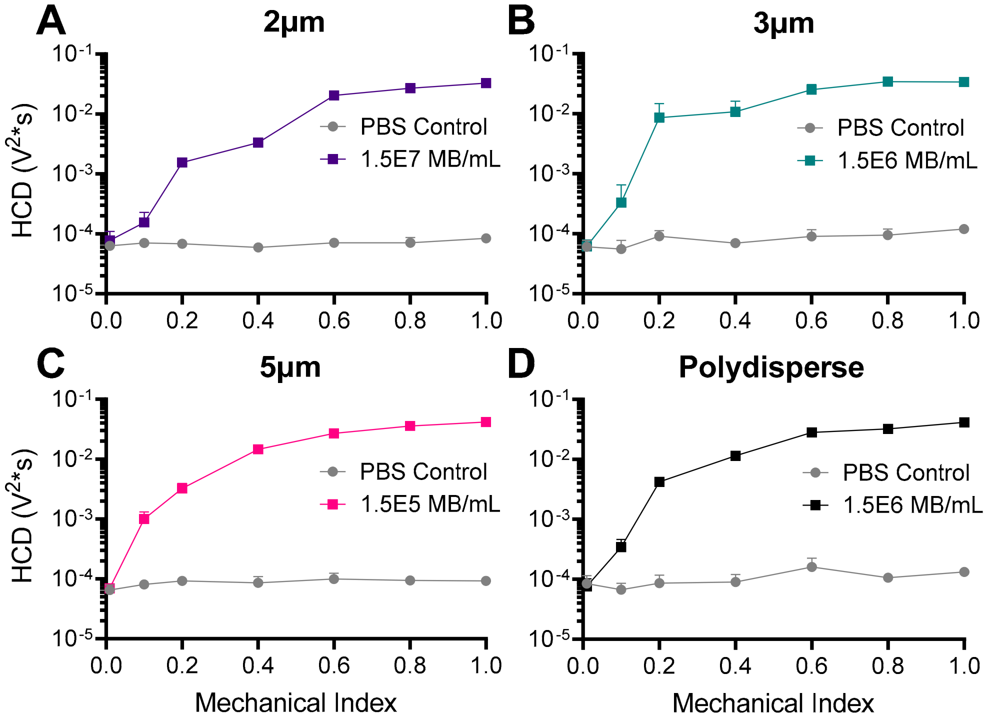

Figure S4. Harmonic Cavitation Dose (HCD) Controls. (**A**) 2μm microbubbles comparing the HCD in the PBS control and the $1.5*10^7$ MBs/mL concentration. (**B**) 3μm microbubbles at $1.5*10^6$ MBs/mL compared to the PBS control. (**C**) 5μm microbubbles at $1.5*10^5$ MBs/mL. (**D**) Polydisperse microbubbles at $1.5*10^6$ MBs/mL. The microbubble data is the raw HCD and was not subtracted from the PBS control like other HCD plots. Data represents the mean and errors bars show standard deviation (N = 4).

Table S1. Statistical Analysis of HCD vs MI. Table shows the adjusted *p*-values obtained when performing a Tukey's multiple comparisons test. Test was conducted by comparing each mechanical index value with itself at each distinct microbubble concentration and microbubble size distribution. *p*-values were rounded to four significant digits. Pink-Cyan color scale was used to represent range of *p*-values relative to each other (Pink is higher and Cyan is lower *p*-values).

Adjusted P-Values - Tukey's multiple comparisons test									
Mechanical Index Comparisons	2um				3um				
	Concentration				Concentration				
	1.5×10^{5}	1.5×10^{6}	1.5×10^{7}	1.5×10^{8}	1.5×10^{4}	1.5×10^{5}	1.5×10^{6}	1.5×10^{7}	1.5×10^{8}
0.01 vs. 0.1	0.9999	0.9999	0.9999	0.9999	0.9999	0.9999	0.9999	0.9999	0.9999
0.01 vs. 0.2	0.9999	0.0088	0.9525	0.9999	0.9999	0.9988	0.113	0.9999	0.9999
0.01 vs. 0.4	0.9999	0.0097	0.2989	0.9999	0.9999	0.995	0.017	0.9878	0.9999
0.01 vs. 0.6	0.9532	0.0001	0.0001	0.0001	0.9996	0.002	0.0001	0.0006	0.7417
0.01 vs. 0.8	0.9567	0.0001	0.0001	0.0001	0.9572	0.0001	0.0001	0.0001	0.0554
0.01 vs. 1.0	0.3522	0.0001	0.0001	0.0001	0.7023	0.0001	0.0001	0.0001	0.0407
0.1 vs. 0.2	0.9999	0.0087	0.9624	0.9999	0.9999	0.9989	0.1374	0.9999	0.9999
0.1 vs. 0.4	0.9999	0.0095	0.3243	0.9999	0.9999	0.9953	0.022	0.9879	0.9999
0.1 vs. 0.6	0.952	0.0001	0.0001	0.0001	0.9996	0.002	0.0001	0.0006	0.7421
0.1 vs. 0.8	0.9555	0.0001	0.0001	0.0001	0.9578	0.0001	0.0001	0.0001	0.0555
0.1 vs. 1.0	0.3491	0.0001	0.0001	0.0001	0.7039	0.0001	0.0001	0.0001	0.0408
0.2 vs. 0.4	0.9999	0.9999	0.8867	0.9999	0.9999	0.9999	0.9924	0.9895	0.9999
0.2 vs. 0.6	0.9874	0.0001	0.0001	0.0001	0.9999	0.0107	0.0001	0.0007	0.7386
0.2 vs. 0.8	0.9887	0.0001	0.0001	0.0001	0.9714	0.0001	0.0001	0.0001	0.0546
0.2 vs. 1.0	0.4944	0.0001	0.0001	0.0001	0.7495	0.0001	0.0001	0.0001	0.0401
0.4 vs. 0.6	0.9761	0.0001	0.0001	0.0001	0.9998	0.0169	0.0004	0.0091	0.8132
0.4 vs. 0.8	0.9782	0.0001	0.0001	0.0001	0.9681	0.0001	0.0001	0.0001	0.0771
0.4 vs. 1.0	0.4288	0.0001	0.0001	0.0001	0.7374	0.0001	0.0001	0.0001	0.0575
0.6 vs. 0.8	0.9999	0.0001	0.0006	0.0029	0.9976	0.6391	0.0668	0.1053	0.7653
0.6 vs. 1.0	0.9202	0.0001	0.0001	0.0001	0.9095	0.0376	0.0859	0.0689	0.6968
0.8 vs. 1.0	0.9151	0.9798	0.0043	0.4407	0.997	0.7772	0.9999	0.9999	0.9999
Mechanical Index Comparisons	5um				Polydisperse				
	Concentration				Concentration				
	1.5×10^{4}	1.5×10^{5}	1.5×10^{6}	1.5×10^{7}	1.5×10^{4}	1.5×10^{5}	1.5×10^{6}	1.5×10^{7}	1.5×10^{8}
0.01 vs. 0.1	0.9999	0.9857	0.9999	0.9999	0.9999	0.9999	0.9999	0.9999	0.9999
0.01 vs. 0.2	0.9999	0.1171	0.0573	0.9999	0.9999	0.9987	0.3035	0.9999	0.9999
0.01 vs. 0.4	0.9999	0.0001	0.0001	0.8206	0.9999	0.9882	0.0001	0.9948	0.9999
0.01 vs. 0.6	0.9768	0.0001	0.0001	0.0001	0.9999	0.0914	0.0001	0.0001	0.0003
0.01 vs. 0.8	0.0001	0.0001	0.0001	0.0001	0.9999	0.0003	0.0001	0.0001	0.0001
0.01 vs. 1.0	0.0001	0.0001	0.0001	0.0001	0.9991	0.0001	0.0001	0.0001	0.0001
0.1 vs. 0.2	0.9999	0.4869	0.1237	0.9999	0.9999	0.999	0.3903	0.9999	0.9999
0.1 vs. 0.4	0.9999	0.0001	0.0001	0.8185	0.9999	0.9897	0.0001	0.9956	0.9999
0.1 vs. 0.6	0.9774	0.0001	0.0001	0.0001	0.9999	0.0956	0.0001	0.0001	0.0004
0.1 vs. 0.8	0.0467	0.0001	0.0001	0.0001	0.9999	0.0003	0.0001	0.0001	0.0001
0.1 vs. 1.0	0.0001	0.0001	0.0001	0.0001	0.9991	0.0001	0.0001	0.0001	0.0001
0.2 vs. 0.4	0.9999	0.0001	0.0001	0.8202	0.9999	0.9999	0.0039	0.9993	0.9999
0.2 vs. 0.6	0.9903	0.0001	0.0001	0.0001	0.9999	0.2713	0.0001	0.0001	0.0004
0.2 vs. 0.8	0.0652	0.0001	0.0001	0.0001	0.9999	0.0018	0.0001	0.0001	0.0001
0.2 vs. 1.0	0.0001	0.0001	0.0001	0.0001	0.9993	0.0001	0.0001	0.0001	0.0001
0.4 vs. 0.6	0.9909	0.0001	0.0001	0.0001	0.9999	0.4135	0.0001	0.0001	0.0009
0.4 vs. 0.8	0.0666	0.0001	0.0001	0.0001	0.9999	0.0042	0.0001	0.0001	0.0001
0.4 vs. 1.0	0.0001	0.0001	0.0001	0.0001	0.9994	0.0001	0.0001	0.0001	0.0001

0.6 vs. 0.8	0.3072	0.0001	0.0001	0.0001	0.9999	0.5511	0.2515	0.0011	0.0102
0.6 vs. 1.0	0.0003	0.0001	0.0001	0.0001	0.9999	0.0998	0.0001	0.0001	0.0001
0.8 vs. 1.0	0.1898	0.0001	0.0001	0.0005	0.9999	0.966	0.0001	0.0266	0.5542

Table S2. Statistical Analysis of BCD vs MI. Table shows the adjusted p-values obtained when performing a Tukey's multiple comparisons test. Test was conducted by comparing each mechanical index value with itself at each distinct microbubble concentration and microbubble size distribution. P-values were rounded to four significant digits. Pink-Cyan color scale was used to represent range of P-values relative to each other (Pink is higher and Cyan is lower P-values).

Adjusted P-Values - Tukey's multiple comparisons test									
Mechanical Index Comparisons	2um				3um				
	Concentration				Concentration				
	1.5 × 10^5	1.5 × 10^6	1.5 × 10^7	1.5 × 10^8	1.5 × 10^4	1.5 × 10^5	1.5 × 10^6	1.5 × 10^7	1.5 ×10^8
0.01 vs. 0.1	0.998	0.9647	0.9993	0.9981	0.9999	0.9999	0.9999	0.9999	0.9999
0.01 vs. 0.2	0.9999	0.998	0.9999	0.9994	0.9999	0.9999	0.9999	0.9999	0.9999
0.01 vs. 0.4	0.9999	0.9999	0.9974	0.9998	0.9999	0.9999	0.9988	0.9688	0.9961
0.01 vs. 0.6	0.9999	0.1544	0.0001	0.0001	0.9999	0.9999	0.0048	0.0001	0.0002
0.01 vs. 0.8	0.9985	0.0001	0.0001	0.0001	0.9999	0.9979	0.0001	0.0001	0.0001
0.01 vs. 1.0	0.9999	0.0001	0.0001	0.0001	0.9999	0.9993	0.0001	0.0001	0.0001
0.1 vs. 0.2	0.9999	0.9997	0.9999	0.9999	0.9999	0.9999	0.9999	0.9999	0.9999
0.1 vs. 0.4	0.9917	0.8778	0.9459	0.9691	0.9999	0.9999	0.9995	0.9742	0.9971
0.1 vs. 0.6	0.9981	0.013	0.0001	0.0001	0.9999	0.9999	0.0061	0.0001	0.0003
0.1 vs. 0.8	0.9359	0.0001	0.0001	0.0001	0.9999	0.9984	0.0001	0.0001	0.0001
0.1 vs. 1.0	0.9897	0.0001	0.0001	0.0001	0.9999	0.9995	0.0001	0.0001	0.0001
0.2 vs. 0.4	0.9995	0.9794	0.9834	0.9823	0.9999	0.9999	0.9997	0.9777	0.998
0.2 vs. 0.6	0.9999	0.0404	0.0001	0.0001	0.9999	0.9999	0.0068	0.0001	0.0003
0.2 vs. 0.8	0.986	0.0001	0.0001	0.0001	0.9999	0.9992	0.0001	0.0001	0.0001
0.2 vs. 1.0	0.9993	0.0001	0.0001	0.0001	0.9999	0.9998	0.0001	0.0001	0.0001
0.4 vs. 0.6	0.9999	0.2774	0.0001	0.0001	0.9999	0.9999	0.0234	0.0001	0.0025
0.4 vs. 0.8	0.9998	0.0001	0.0001	0.0001	0.9999	0.9989	0.0001	0.0001	0.0001
0.4 vs. 1.0	0.9999	0.0001	0.0001	0.0001	0.9999	0.9997	0.0001	0.0001	0.0001
0.6 vs. 0.8	0.9984	0.0001	0.0001	0.0001	0.9999	0.9999	0.001	0.0001	0.006
0.6 vs. 1.0	0.9999	0.0001	0.0001	0.0001	0.9999	0.9999	0.0001	0.0001	0.0001
0.8 vs. 1.0	0.9999	0.0111	0.0001	0.0001	0.9999	0.9999	0.091	0.0001	0.0227
Mechanical Index Comparisons	5um				Polydisperse				
	Concentration				Concentration				
	1.5 × 10^4	1.5 × 10^5	1.5 × 10^6	1.5 × 10^7	1.5 × 10^4	1.5 × 10^5	1.5 × 10^6	1.5 × 10^7	1.5 ×10^8
0.01 vs. 0.1	0.9999	0.9999	0.9999	0.9999	0.9999	0.9999	0.9997	0.9999	0.9999
0.01 vs. 0.2	0.9997	0.9999	0.9999	0.9999	0.9996	0.9999	0.9931	0.9999	0.998
0.01 vs. 0.4	0.9999	0.9999	0.9498	0.7319	0.9999	0.9999	0.4065	0.9836	0.9747
0.01 vs. 0.6	0.9999	0.9999	0.0001	0.0001	0.9988	0.5766	0.0001	0.0001	0.0001
0.01 vs. 0.8	0.9998	0.9916	0.0001	0.0001	0.9999	0.0502	0.0001	0.0001	0.0001
0.01 vs. 1.0	0.9999	0.4781	0.0001	0.0001	0.9943	0.0053	0.0001	0.0001	0.0001
0.1 vs. 0.2	0.9999	0.9999	0.9999	0.9999	0.9999	0.9999	0.9999	0.9999	0.9997
0.1 vs. 0.4	0.9999	0.9999	0.965	0.652	0.9999	0.9999	0.6557	0.9912	0.9902
0.1 vs. 0.6	0.9999	0.9992	0.0001	0.0001	0.9996	0.703	0.0001	0.0001	0.0001
0.1 vs. 0.8	0.9968	0.963	0.0001	0.0001	0.9999	0.0829	0.0001	0.0001	0.0001
0.1 vs. 1.0	0.9999	0.3333	0.0001	0.0001	0.9977	0.01	0.0001	0.0001	0.0001
0.2 vs. 0.4	0.9995	0.9999	0.9549	0.6653	0.999	0.9999	0.8331	0.9932	0.9999
0.2 vs. 0.6	0.9999	0.9996	0.0001	0.0001	0.9999	0.6869	0.0001	0.0001	0.0001
0.2 vs. 0.8	0.9883	0.9715	0.0001	0.0001	0.9999	0.0777	0.0001	0.0001	0.0001
0.2 vs. 1.0	0.9996	0.3611	0.0001	0.0001	0.9999	0.0092	0.0001	0.0001	0.0001
0.4 vs. 0.6	0.9999	0.9996	0.0001	0.0001	0.9975	0.6419	0.0001	0.0001	0.0001

0.4 vs. 0.8	0.9999	0.973	0.0001	0.0001	0.9999	0.0651	0.0001	0.0001	0.0001
0.4 vs. 1.0	0.9999	0.3667	0.0001	0.0001	0.9906	0.0073	0.0001	0.0001	0.0001
0.6 vs. 0.8	0.996	0.999	0.0001	0.0001	0.9998	0.8754	0.0001	0.0001	0.0001
0.6 vs. 1.0	0.9999	0.6297	0.0001	0.0001	0.9999	0.4306	0.0001	0.0001	0.0001
0.8 vs. 1.0	0.9998	0.8918	0.0001	0.0001	0.9985	0.9896	0.0001	0.0001	0.0001

Table S3. Statistical Analysis of HCD vs Number Concentration. Table shows the adjusted p-values obtained when performing a Tukey's multiple comparisons test. Test was conducted by comparing each number concentration value with itself at each distinct mechanical index and microbubble size distribution. P-values were rounded to four significant digits. Pink-Cyan color scale was used to represent range of P-values relative to each other (Pink is higher and Cyan is lower P-values).

2um - Adjusted P-Values - Tukey's multiple comparisons test							
Concentration Comparisons	Mechanical Index						
	0.01	0.10	0.20	0.40	0.60	0.80	1.00
1.5×10^5 vs. 1.5×10^6	0.9999	0.9999	0.0058	0.0046	0.0001	0.0001	0.0001
1.5×10^5 vs. 1.5×10^7	0.9999	0.9999	0.8685	0.1664	0.0001	0.0001	0.0001
1.5×10^5 vs. 1.5×10^8	0.9999	0.9999	0.9973	0.9999	0.0001	0.0001	0.0001
1.5×10^6 vs. 1.5×10^7	0.9999	0.9999	0.05	0.5177	0.0442	0.9999	0.0163
1.5×10^6 vs. 1.5×10^8	0.9999	0.9999	0.0031	0.0055	0.0001	0.0001	0.0001
1.5×10^7 vs. 1.5×10^8	0.9999	0.9999	0.7698	0.1852	0.0001	0.0001	0.0001
3um - Adjusted P-Values - Tukey's multiple comparisons test							
1.5×10^4 vs. 1.5×10^5	0.9999	0.9999	0.9932	0.9765	0.0041	0.0001	0.0001
1.5×10^4 vs. 1.5×10^6	0.9999	0.9999	0.0767	0.0105	0.0001	0.0001	0.0001
1.5×10^4 vs. 1.5×10^7	0.9999	0.9999	0.9999	0.9543	0.0014	0.0001	0.0001
1.5×10^4 vs. 1.5×10^8	0.9999	0.9999	0.9999	0.9999	0.8117	0.279	0.5564
1.5×10^5 vs. 1.5×10^6	0.9999	0.9999	0.1945	0.0554	0.002	0.0001	0.0058
1.5×10^5 vs. 1.5×10^7	0.9999	0.9999	0.9892	0.9999	0.9978	0.6534	0.9999
1.5×10^5 vs. 1.5×10^8	0.9999	0.9999	0.9867	0.9854	0.0926	0.0563	0.0009
1.5×10^6 vs. 1.5×10^7	0.9999	0.9999	0.0671	0.0745	0.0058	0.0032	0.0077
1.5×10^6 vs. 1.5×10^8	0.9999	0.9999	0.0625	0.013	0.0001	0.0001	0.0001
1.5×10^7 vs. 1.5×10^8	0.9999	0.9999	0.9999	0.9687	0.0419	0.0008	0.0006
5um - Adjusted P-Values - Tukey's multiple comparisons test							
1.5×10^4 vs. 1.5×10^5	0.9999	0.8325	0.0393	0.0001	0.0001	0.0001	0.0001
1.5×10^4 vs. 1.5×10^6	0.9999	0.9871	0.0166	0.0001	0.0001	0.0001	0.0001
1.5×10^4 vs. 1.5×10^7	0.9999	0.9999	0.9965	0.5933	0.0001	0.0001	0.0001
1.5×10^5 vs. 1.5×10^6	0.9999	0.9559	0.9888	0.001	0.954	0.3727	0.0001
1.5×10^5 vs. 1.5×10^7	0.9999	0.8237	0.0222	0.0001	0.0001	0.0001	0.0001
1.5×10^6 vs. 1.5×10^7	0.9999	0.9851	0.0089	0.0001	0.0001	0.0001	0.0001
Polydisperse - Adjusted P-Values - Tukey's multiple comparisons test							
1.5×10^4 vs. 1.5×10^5	0.9999	0.9999	0.9889	0.9512	0.1085	0.0004	0.0001
1.5×10^4 vs. 1.5×10^6	0.9999	0.9999	0.1979	0.0001	0.0001	0.0001	0.0001
1.5×10^4 vs. 1.5×10^7	0.9999	0.9999	0.9998	0.973	0.0001	0.0001	0.0001
1.5×10^4 vs. 1.5×10^8	0.9999	0.9999	0.9999	0.9994	0.0006	0.0001	0.0001
1.5×10^5 vs. 1.5×10^6	0.9999	0.9999	0.4434	0.0001	0.0001	0.0001	0.0001
1.5×10^5 vs. 1.5×10^7	0.9999	0.9999	0.998	0.9999	0.0099	0.0001	0.0001
1.5×10^5 vs. 1.5×10^8	0.9999	0.9999	0.9869	0.9875	0.4206	0.0066	0.0003
1.5×10^6 vs. 1.5×10^7	0.9999	0.9999	0.2726	0.0001	0.0001	0.0001	0.0001

1.5 × 10^6 vs. 1.5 × 10^8	0.9999	0.9999	0.1897	0.0001	0.0001	0.0001	0.0001
1.5 × 10^7 vs. 1.5 × 10^8	0.9999	0.9999	0.9997	0.9952	0.4924	0.1623	0.003

Table S4. Statistical Analysis of BCD vs Number Concentration. Table shows the adjusted p-values obtained when performing a Tukey's multiple comparisons test. Test was conducted by comparing each number concentration value with itself at each distinct mechanical index and microbubble size distribution. P-values were rounded to four significant digits. Pink-Cyan color scale was used to represent range of P-values relative to each other (Pink is higher and Cyan is lower P-values).

2um - Adjusted P-Values - Tukey's multiple comparisons test							
Concentration Comparisons	Mechanical Index						
	0.01	0.10	0.20	0.40	0.60	0.80	1.00
1.5 × 10^5 vs. 1.5 × 10^6	0.9996	0.9918	0.998	0.9951	0.0449	0.0001	0.0001
1.5 × 10^5 vs. 1.5 × 10^7	0.9993	0.9999	0.9997	0.9918	0.0001	0.0001	0.0001
1.5 × 10^5 vs. 1.5 × 10^8	0.9993	0.9994	0.9898	0.9998	0.0001	0.0001	0.0001
1.5 × 10^6 vs. 1.5 × 10^7	0.9955	0.9942	0.9998	0.9999	0.0001	0.0001	0.0001
1.5 × 10^6 vs. 1.5 × 10^8	0.9956	0.9984	0.9992	0.9987	0.0001	0.0001	0.0001
1.5 × 10^7 vs. 1.5 × 10^8	0.9999	0.9998	0.9967	0.9971	0.0468	0.0001	0.0001
3um - Adjusted P-Values - Tukey's multiple comparisons test							
1.5 × 10^4 vs. 1.5 × 10^5	0.9999	0.9999	0.9999	0.9999	0.9989	0.9883	0.9964
1.5 × 10^4 vs. 1.5 × 10^6	0.9999	0.9999	0.9999	0.9883	0.003	0.0001	0.0001
1.5 × 10^4 vs. 1.5 × 10^7	0.9999	0.9999	0.9999	0.8936	0.0001	0.0001	0.0001
1.5 × 10^4 vs. 1.5 × 10^8	0.9999	0.9999	0.9999	0.9795	0.0002	0.0001	0.0001
1.5 × 10^5 vs. 1.5 × 10^6	0.9999	0.9999	0.9999	0.9917	0.0072	0.0001	0.0001
1.5 × 10^5 vs. 1.5 × 10^7	0.9999	0.9999	0.9999	0.9093	0.0001	0.0001	0.0001
1.5 × 10^5 vs. 1.5 × 10^8	0.9999	0.9999	0.9999	0.9847	0.0005	0.0001	0.0001
1.5 × 10^6 vs. 1.5 × 10^7	0.9999	0.9999	0.9999	0.9935	0.1688	0.0017	0.0001
1.5 × 10^6 vs. 1.5 × 10^8	0.9999	0.9999	0.9999	0.9999	0.9379	0.9991	0.9393
1.5 × 10^7 vs. 1.5 × 10^8	0.9999	0.9999	0.9999	0.9971	0.5777	0.0041	0.0001
5um - Adjusted P-Values - Tukey's multiple comparisons test							
1.5 × 10^4 vs. 1.5 × 10^5	0.9988	0.9986	0.9999	0.9837	0.9867	0.9972	0.3051
1.5 × 10^4 vs. 1.5 × 10^6	0.9876	0.9999	0.9998	0.9146	0.0001	0.0001	0.0001
1.5 × 10^4 vs. 1.5 × 10^7	0.9953	0.9992	0.9999	0.5953	0.0001	0.0001	0.0001
1.5 × 10^5 vs. 1.5 × 10^6	0.9979	0.9991	0.9999	0.742	0.0001	0.0001	0.0001
1.5 × 10^5 vs. 1.5 × 10^7	0.9998	0.9999	0.9999	0.3762	0.0001	0.0001	0.0001
1.5 × 10^6 vs. 1.5 × 10^7	0.9997	0.9995	0.9999	0.9315	0.0177	0.2633	0.9915
Polydisperse - Adjusted P-Values - Tukey's multiple comparisons test							
1.5 × 10^4 vs. 1.5 × 10^5	0.9999	0.9999	0.9987	0.9999	0.7321	0.0427	0.0231
1.5 × 10^4 vs. 1.5 × 10^6	0.9986	0.9999	0.9999	0.3758	0.0001	0.0001	0.0001
1.5 × 10^4 vs. 1.5 × 10^7	0.9999	0.9999	0.9968	0.923	0.0001	0.0001	0.0001
1.5 × 10^4 vs. 1.5 × 10^8	0.9977	0.9988	0.9997	0.9707	0.0001	0.0001	0.0001
1.5 × 10^5 vs. 1.5 × 10^6	0.999	0.9999	0.999	0.4589	0.0001	0.0001	0.0001
1.5 × 10^5 vs. 1.5 × 10^7	0.9999	0.9999	0.9999	0.9595	0.0001	0.0001	0.0001
1.5 × 10^5 vs. 1.5 × 10^8	0.9983	0.997	0.9999	0.9885	0.0001	0.0001	0.0001
1.5 × 10^6 vs. 1.5 × 10^7	0.9994	0.9999	0.9974	0.8625	0.9755	0.0038	0.0189
1.5 × 10^6 vs. 1.5 × 10^8	0.9999	0.9988	0.9998	0.7625	0.4798	0.0911	0.0001
1.5 × 10^7 vs. 1.5 × 10^8	0.9988	0.9995	0.9999	0.9997	0.1774	0.0001	0.0001
